# Adaptation to Ephemeral Habitat May Overcome Natural Barriers and Severe Habitat Fragmentation in a Fire-Dependent Species, the Bachman's Sparrow (*Peucaea aestivalis*)

**DOI:** 10.1371/journal.pone.0105782

**Published:** 2014-09-02

**Authors:** Blain Cerame, James A. Cox, Robb T. Brumfield, James W. Tucker, Sabrina S. Taylor

**Affiliations:** 1 School of Renewable Natural Resources, Louisiana State University Agricultural Center, Baton Rouge, Louisiana, United States of America; 2 Tall Timbers Research Station and Land Conservancy, Tallahassee, Florida, United States of America; 3 Museum of Natural Science and Department of Biological Sciences, Louisiana State University, Baton Rouge, Louisiana, United States of America; 4 Archbold Biological Station, Venus, Florida, United States of America; University of British Columbia Okanagan, Canada

## Abstract

Bachman's Sparrow (*Peucaea aestivalis*) is a fire-dependent species that has undergone range-wide population declines in recent decades. We examined genetic diversity in Bachman's Sparrows to determine whether natural barriers have led to distinct population units and to assess the effect of anthropogenic habitat loss and fragmentation. Genetic diversity was examined across the geographic range by genotyping 226 individuals at 18 microsatellite loci and sequencing 48 individuals at mitochondrial and nuclear genes. Multiple analyses consistently demonstrated little genetic structure and high levels of genetic variation, suggesting that populations are panmictic. Based on these genetic data, separate management units/subspecies designations or translocations to promote gene flow among fragmented populations do not appear to be necessary. Panmixia in Bachman's Sparrow may be a consequence of an historical range expansion and retraction. Alternatively, high vagility in Bachman's Sparrow may be an adaptation to the ephemeral, fire-mediated habitat that this species prefers. In recent times, high vagility also appears to have offset inbreeding and loss of genetic diversity in highly fragmented habitat.

## Introduction

Genetic structure in wildlife populations is typically assessed with respect to natural barriers or anthropogenic habitat loss and fragmentation. Fragmented habitats created by natural barriers, such as rivers, oceans, deserts and mountain ranges, have documented major effects on population differentiation [1] and species-level diversity [2]–[5]. For example, in the southeastern US, the Apalachicola, Tombigbee, and Mississippi Rivers are associated with genetic differentiation in several taxa, ranging from vertebrates to plants [6]–[12]. Population differentiation caused by natural barriers is important to identify because it may produce distinct lineages that warrant attention to ensure maintenance of biodiversity.

In addition to natural habitat fragmentation, recent anthropogenic habitat fragmentation, degradation and loss also have the potential to disrupt gene flow among populations [13]. Many species that were historically distributed across broad geographic areas have become restricted to increasingly smaller and more isolated patches, creating habitat islands that may bottleneck remaining populations and prevent genetic contact among them [14]. As population size decreases, genetic drift and inbreeding increase, potentially leading to reduced fitness as a result of loss of alleles, expression of deleterious recessive alleles, or loss of heterozygote advantage [13], [15], [16]. Estimating genetic variation and inbreeding in habitat fragments is important because it can help to identify populations that may require management actions such as translocations to promote gene flow and protect evolutionary potential.

Although natural and anthropogenic fragmentation can shape genetic structure of populations, other underlying natural processes may also influence structure significantly. In particular, species that specialize in ephemeral or disturbed habitat may have dispersal strategies or adaptations that are distinct from or absent in species found in more stable habitats [17]. For instance, species adapted to fire-mediated habitat may depend on early, and ephemeral, successional stages, which may require high vagility to colonize newly burned habitat and abandon habitat that has become unsuitable. Fire has a significant effect on gene flow in several species occupying fire-mediated habitat [17]–[20]; however, the effects of fire-mediated landscape change on evolutionary processes are poorly studied despite their potentially strong influence [17], [18].

**Figure 1:**
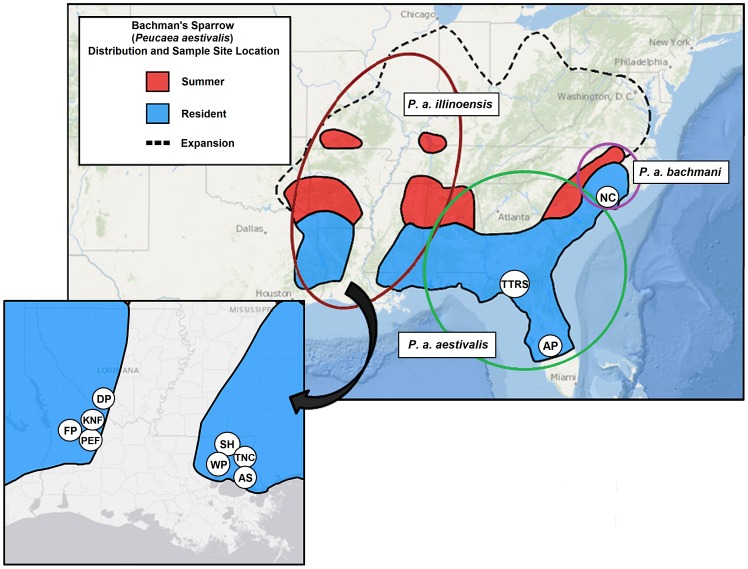
Bachman's Sparrow distribution including historic range expansion and subspecific ranges. Ranges as described by the AOU [21] and Dunning [74]. Sampling locations include: Fort Polk WMA (FP), Palustris Experimental Forest (PEF), Kisatchie National Forest (KNF), Dry Prong WMA (DP), Camp Whispering Pines (WP), Sandy Hollow WMA (SH), Talisheek Pines Wetland Preserve (TNC), Abita Springs (AS), Tall Timbers Research Station (TTRS), Avon Park Air Force Range (AP), and North Carolina (NC).

**Figure 2:**
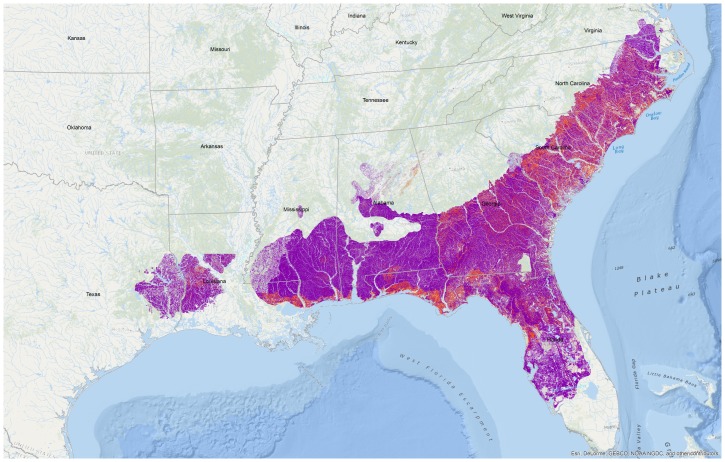
Historic (purple) and current (red) longleaf pine habitat in the southeastern US. GIS data provided by NatureServe and LandScope America.

In the southeastern US, longleaf pine (*Pinus palustris*) forests are a fire-mediated ecosystem with several closely associated plant and animal species. Population structure in one species, the Bachman's Sparrow (*Peucaea aestivalis*), is potentially complex because it is influenced by natural and anthropogenic fragmentation as well as ephemeral, fire-mediated habitat preferences. The sparrow currently consists of three subspecies [21] (Figure 1): *P. a. illinoensis* occupies the northern and westernmost areas of Bachman's Sparrow range including Texas, Louisiana, Indiana, Illinois and Missouri; *P. a. aestivalis* occupies areas east into Florida, Georgia and South Carolina; and *P. a. bachmani* occupies North Carolina and Virginia [21] (Figure 1). In contrast, Sibley [22] points to morphological differences between individuals on either side of the Mississippi River, so distinct populations may be more appropriately delineated by natural barriers: not only is the Mississippi River itself a major geological barrier, but its vast adjacent bayous and swamps bisect the longleaf pine habitat preferred by Bachman's Sparrow (Figure 2). Despite groupings by the American Ornithologists' Union [21] and Sibley [22], no genetic data exist for population structure in Bachman's Sparrows, data that might help to identify genuinely distinct populations that warrant conservation and management efforts.

In addition to natural barriers, loss (over 95%) and fragmentation of longleaf pine habitat [23] (Figure 2) has caused population declines and a fragmented distribution in Bachman's Sparrows, factors that could restrict gene flow. However, Bachman's Sparrows also move frequently because post-fire plant growth can eliminate preferred habitat structure within two years following a fire [24]–[28]. Accordingly, sparrows may have high dispersal rates as an adaptation to ephemeral habitat.

Bachman's Sparrow is listed as a species of conservation concern both internationally (IUCN) as well as within every state in which it breeds [29]. Therefore, quantifying genetic structure and diversity is important for identifying and conserving distinct genetic lineages as well as understanding the effects of habitat fragmentation on genetic diversity and gene flow. In addition, broad-scale genetic assessments could help clarify the influence of historic disturbance processes (fire) on adaptation to disturbance stemming from recent habitat fragmentation.

The objectives of this study are to: 1) examine genetic structure and diversity in a species adapted to natural disturbances caused by fire; 2) examine Bachman's Sparrow population differentiation across its range to evaluate whether current subspecies designations are valid; and 3) evaluate gene flow among and genetic diversity within habitat fragments to identify areas of restricted gene flow and populations with inbreeding and low levels of genetic diversity. The results of this study should help to ensure that populations of high genetic value are conserved, that genetic variation is maintained and inbreeding depression is reduced in remnant populations, and finally, provide a better understanding of the effect of ephemeral habitat on gene flow.

## Materials and Methods

This study was carried out in strict accordance with the recommendations in the Guide for the Care and Use of Laboratory Animals of the National Institutes of Health. Protocols were approved by the Institutional Animal Care and Use Committee of the Louisiana State University AgCenter (Permit Numbers: AE2011-04 and A2012-05) and Tall Timbers Research Station (Permit Number: VE-2002-01). Birds were banded and bled under Federal Bird Banding Permits 07732, 22648 & 24466, State Permits FFWCC LSSC-05-0205 & 29-wmb-02-143 (Florida) and LNHP-11-062 & LNHP-12-023 (Louisiana), Wildlife Management Area Permit WL-Research-2011-03 (Louisiana), and US Department of Agriculture Forest Service Permit 2610 (Kisatchie National Forest, Louisiana).

### Study Sites and Field Protocols

Sampling in Louisiana was conducted in areas with recent Ebird records and separated by the Mississippi River, a potentially important geographic barrier to dispersal. We sampled four sites on the west and three sites on the east side of the Mississippi River (Figure 1). Western Louisiana has larger, contiguous longleaf pine tracts whereas eastern Louisiana has smaller and more fragmented patches of longleaf pine. Louisiana populations were sampled from February through June in 2011 (n = 26) and 2012 (n = 88) on public and private lands. Sampling across the broader geographic range was conducted using vouchered Louisiana (n = 30), North Carolina (n = 3), and Florida (n = 1) tissue samples from the Collection of Genetic Resources at the Louisiana State University Museum of Natural Science, and in association with long-term research projects in Florida focused on Bachman's Sparrows [26], [30], which included blood samples from the Tall Timbers Research Station (hereafter Tall Timbers; n = 32 sampled in 2011) and Avon Park Air Force Range (hereafter Avon Park; n = 47 sampled in 2003 and 2004) (Figure 1, Table 1). Individuals (excluding LSU Museum of Natural Science samples) were captured with mist nests using conspecific playbacks [31], banded with a federal band, and bled (<100 µl) via venipuncture of the brachial vein. Blood samples were stored in 1.0 mL of Queen's lysis buffer [32] at 10°C until they could be processed. Hand-held GPS units with <10 m precision were used to geographically reference capture locations.

**Table 1:** Study site, geographic location, ownership and managing entity, provenance, and sample size for Bachman's Sparrow populations.

Study Site	Location	Ownership & Managing Bodies	Provenance and Sample Size (*n*)
Fort Polk WMA^1^	Vernon Parish, LA; Calcasieu Ranger District, KNF^2^	U.S Army; U.S. Forest Service; LDWF^3^	Field = 25
Dry Prong	Grant Parish, LA; Catahoula Ranger District, KNF^2^	U.S. Forest Service	Field = 20 LSUMZ^4^ = 5
Kisatchie National Forest	Rapides Parish, LA; Kisatchie Ranger District, KNF^2^	U.S. Forest Service	Field = 14 LSUMZ^4^ = 1
Palustris Experimental Forest	Rapides Parish, LA; Kisatchie Ranger District, KNF^2^	U.S. Forest Service	Field = 10 LSUMZ^4^ = 3
Sandy Hollow WMA^1^	Tangipahoa Parish, LA	Tangipahoa School Board; LDWF	Field = 23 LSUMZ^4^ = 6
Lee Memorial Forest	Washington Parish, LA	Louisiana State University Agricultural Center	Field = 2
Camp Whispering Pines	Tangipahoa Parish, LA	Girl Scouts of the USA	Field = 14
Talisheek Pine Wetlands Preserve	St. Tammany Parish, LA	Money Hill Real Estate Group; TNC^5^	Field = 5
Abita Springs	St. Tammany Parish, LA		LSUMZ^4^ = 15
Florida	Madison County, FL		LSUMZ^4^ = 1
North Carolina	Brunswick and Columbus County, NC		LSUMZ^4^ = 3
Tall Timbers Land Conservancy and Research Station	Leon County, FL	Tall Timbers Land Conservancy	Field = 32
Avon Park Air Force Range	Polk and Highlands County, FL	U.S. Air Force	Field = 47

1 Wildlife Management Area.

2 Kisatchie National Forest.

3 Louisiana Department of Wildlife and Fisheries.

4 Louisiana State University Museum of Natural Science.

5 The Nature Conservancy.

### Molecular Methods

Total DNA was extracted from blood (n = 226) using DNeasy Blood and Tissue kits (Qiagen, Valencia, CA). Samples were amplified using polymerase chain reaction (PCR) with an Eppendorf Mastercycler pro S thermal cycler. Nuclear microsatellite primer pairs (n = 23) developed in other avian species were tested, and 19 amplified successfully (Table S1). PCRs consisted of 1.0 µl DNA, 1X buffer, 2.0 mM MgCl_2_, 0.8 mM dNTPs, 0.10 µM each of forward and reverse primers, 0.5 µl of 100% dimethyl sulfoxide (DMSO), 1 M betaine, 0.03 µM M13 fluorescent tag, 2.0 units Taq DNA polymerase (New England BioLabs, Ipswich, MA), and nanopure water to a final volume of 10 µl. PCR amplification conditions were 95°C for 30 seconds followed by 34 cycles of 95°C for 1 minute; 48–60°C (see Table S1) for 1 minute, 72°C for 1 minute and a final extension step of 72°C for 4 minutes. Forward or reverse primers were labeled at the 5′ end with M13 tags (LI-Cor Biosciences) to allow the DNA amplicons to be detected by infrared laser fluorescence. For each amplified sample, 0.8 µl of product was resolved by electrophoresis on a 25-cm, 7% polyacrylamide gel and genotyped on a LI-Cor 4200 Gene ReadIR DNA Analyzer (LI-Cor Biosciences) with 50–350 bp IRDye 700 and 800 frequency size standards (LI-Cor Biosciences). In conjunction with the size standards, samples representing all allele sizes for each locus were added to gels as additional size markers to ensure consistent genotyping. Allele sizes were estimated using Saga v. 3.2 (LI-Cor Biosciences) and verified visually.

Sequence data were obtained for one mitochondrial locus, the nicotinamide adenine dinucleotide dehydrogenase subunit 2 (ND2) using primer L5215 from [33] and H6313 from [34], and one nuclear locus, the transforming growth factor β-2 intron 5 (TGFβ2) using primers from [35]. Both genes were sequenced for 15 individuals each from Tall Timbers (north Florida), Avon Park (south Florida), and eastern and western populations in Louisiana. Three individuals from Columbus County (North Carolina) were also sequenced at these genes. PCRs consisted of 1 µl DNA, 1X buffer, 1.50 mM MgCl_2_, 0.8 mM of dNTPs, 1.25 µM of each forward and reverse primers, 2.5 units Taq DNA polymerase (New England BioLabs, Ipswich, MA), and nanopure water for a final volume of 25 µl. PCR amplification conditions were as follows: 95°C for 30 seconds followed by 34 cycles of 94°C for 30 seconds, 50°C (ND2)/60°C (TGFβ2) for 30 seconds, 72°C for 1 minute, and a final extension step of 72°C for 7 minutes. PCR products were sent to Beckman Coulter Laboratories (Danvers, MA) for Sanger single-pass sequencing. Forward and reverse strands were aligned for each sample and corrected using Sequencher v. 5.0 (Gene Codes Corp.).

### Data Analysis

#### Population molecular variation

Microsatellite data were checked for genotyping errors using Microchecker v. 2.2.3 [36]. Hardy-Weinberg Equilibrium (HWE) and linkage disequilibrium were assessed using Genepop v. 4.1.4 [37], [38]. The small number of samples obtained from Lee Memorial Forest (n = 2) and Madison County, Florida (n = 1) were combined with the nearest sampling locations (Talisheek Pine Wetlands Preserve and Tall Timbers, respectively). Exact P-values for HWE were computed using a complete enumeration method for loci <4 alleles [39] and the Monte Carlo Markov Chain (MCMC) method for loci with >4 alleles [40]. Global deviation from HWE for populations was calculated using the same parameters listed above. Significance values were adjusted using a Bonferroni sequential correction for multiple comparisons [41] to maintain an experiment-wise error rate of α = 0.05.

Population genetic variation was measured as average observed and expected heterozygosity, average number of alleles per locus, and allelic richness with Genetix v. 4.03 [42] and Fstat v. 2.9.3 [43]. Initial allelic richness calculations included all populations; however, small sample sizes from North Carolina and Talisheek Pine Wetlands Preserve, Louisiana, substantially reduced allelic richness across populations, so these two populations were dropped and allelic richness was calculated again for the remaining populations. Genepop was used to calculate *F_IS_*, the inbreeding coefficient [44].

For ND2, a 1038 base pair sequence was obtained for 47 individuals, and for TGFβ2 a 570 base pair sequence was obtained for 43 individuals. Some TGFβ2 sequences were heterozygous, therefore, prior to analyzing sequence data for molecular variation, Bayesian computational inference of TGFβ2 gametic phase was performed using the PHASE module in DnaSP v. 5.10.1 [45]. Calculations were carried out over 1,000 iterations, 10 thinning intervals, and 1,000 burn-in iterations with a model that accounted for recombination. All advanced options used the program's default settings. Nucleotide diversity (π), number of haplotypes, and haplotype diversity [46] were calculated for each population using DnaSP. Estimates of sequence divergence among populations were also calculated using DnaSP, which included the number of net nucleotide substitutions per site among populations (*D_a_*) and the average number of nucleotide substitutions per site among populations (*D_xy_*).

#### Analyses of population genetic structure

Genetic differentiation among the five regions was calculated in Genepop with microsatellite data using global *F_ST_* (θ) as well as pairwise *F_ST_* [44] and *R_ST_* (ρ) [47]. Patterns of population structure were analyzed for all microsatellite data using multiple methods to provide less biased assessments of population structure [48]. We used: (1) a Bayesian clustering approach in Structure v. 2.3.2 [49]; (2) a spatial analysis of molecular variance using Geneland v. 4.0 [50]; and, (3) a multivariate analysis using factorial correspondence analysis (FCA) in Genetix v. 4.05.

Structure assesses whether sampled genotypes are substructured into multiple (*K*>1) clusters or constitute a single population (*K* = 1). We implemented Structure with and without the LocPrior clustering algorithm, which accounts for sampling locations and assumes that assignment probability varies among locations. The LocPrior method is appropriate for detecting weak genetic structure [51]. Twenty runs were conducted for values of *K* ranging from 1–11. Each run had a burn-in of 150,000 followed by 150,000 iterations [52]. Plots of MCMC chains were checked to ensure convergence. The admixture model was used because it assumes that all individuals originated from the admixture of *K* parental populations [49] and that allele frequencies were correlated [53]. Using the output from Structure, the best estimate of the number of clusters (*K*) was determined using log-likelihood ratios from Structure following Evanno et al. [54]. This approach identifies the most likely *K* based on changes in the log probability for successive values of *K*. The most likely *K* suggested by initial runs was reassessed in Structure for an additional 25 runs. Averaged results were then calculated to produce a parameter (*r*) that estimates the information on ancestry provided by sampling location in the LocPrior model. Values of *r*≤1 indicate that the inclusion of sampling locations is informative, whereas values of *r*>>1 imply that location data is uninformative [51].

Genetic structure as calculated by Geneland was implemented in R (v. 3.0). Geneland detects population subdivision and barriers to gene flow using a spatially explicit model that incorporates geographic barriers and boundaries among populations into the analysis of genetic structure [55]. Spatial coordinates are coupled with genetic data to optimize the delineation of subpopulations assuming that more distant populations are more genetically differentiated. Unlike the approach used in Structure, all clustering solutions are not equally probable in Geneland. Instead, spatial distributions are used to infer the number of subpopulations, *K*. Initial runs allowed *K* to vary under the following conditions; 10,000 stored iterations of the Markov chain, maximum rate of Poisson process set at the default value of 100, minimum population number set to a minimum of 1 and a maximum of 11, and the number of thinnings set to 10. The uncertainty of the coordinates was set to zero because GPS coordinates were available for each sample. A Correlated Allele Frequency model, a true Spatial model and a false Null Allele model were used in the analysis. Five independent runs of these three parameters were run for each potential *K*.

FCA was run in Genetix to assess population structure among sampling locations using scores derived from two axes. Isolation by distance (IBD) was tested with IBDWS v. 3.23, which examines the correlation between genetic [56] and geographical distances for each pairwise combination. The correlation between genetic and geographic distances was calculated using a reduced major axis regression (RMA) with 10,000 randomizations [57]. Unlike ordinary least-squares regression, RMA optimizes a “best-fit” line by reducing error for both variables simultaneously [57], [58].

Genetic structure in mitochondrial and nuclear DNA sequence data was examined by calculating estimates of global and pairwise *F_ST_* using an analysis of molecular variance (AMOVA) implemented in Arlequin v. 3.11 [59] using 10,000 randomizations of the data. The significance level was set at p≤0.05 for all tests.

To investigate phylogeographic structuring, relationships among mitochondrial and nuclear DNA haplotypes were constructed using statistical parsimony [60], [61] in TCS v. 1.13 [62]. Haplotype networks were used to provide a better representation of phylogenetic relationships where sequences are very similar and the strength of the historical inferences increase as genetic variation decreases [63]. The program assumes that a single polymorphic site with a single variant allele was derived through a single mutation. The probability of parsimony [64] is calculated for pairwise differences until the probability exceeds the default value of 0.95. The mutational differences determined before 0.95 is reached provide an estimate of the maximum number of mutational connections between pairs of sequences justified by the parsimony criterion. Mega v. 5 [65] was also used to construct neighbor joining trees to visualize the evolutionary relatedness among sampled populations. An unrooted neighbor joining tree was constructed after running 2000 replications of the bootstrap method to test for phylogeny. The Maximum Composite Likelihood substitution model included transitions and transversions with the nucleotide substitution rate set at the default of uniform rates. The mitochondrial and nuclear sequences had no missing nucleotide bases, so the gaps/missing data option was set for complete deletion. All three codon positions were used to build the tree, and after the tree was constructed, nodes with less than 50% support were condensed due to the uncertainty of the branching order.

#### Bottlenecks and Population Connectivity

Evidence for recent population bottlenecks was evaluated with Bottleneck v. 1.2.02 [66], [67]. During bottlenecks, rare alleles are lost more quickly than heterozygosity, which should lead to heterozygosity excess [68]. Two estimates of expected heterozygosity were compared based on (1) allele frequencies (*H_e_*) assuming HWE and (2) the number of alleles and sample size (*H_eq_*) assuming mutation-drift equilibrium. Both estimates should be similar at equilibrium, but *H_eq_* will decrease faster than *H_e_* if a population experiences a bottleneck. On the other hand, population expansion would be expected if *H_e_* decreased faster than *H_eq_*. Estimates of heterozygosity were calculated using a two-phase model that requires two parameters: (1) the percentage of mutations that follow a strict stepwise mutational process, and; (2) the variance in size of multistep mutations [67]. Recent research on mutational dynamics in avian microsatellites suggest ∼60% to 80% of mutations involve a single-step change [69], [70]. For this reason, we set the stepwise mutation rate at 70%, used a more conservative value (30%) for multistep mutations, and then ran the analysis using 10,000 iterations. We used the Wilcoxon signed-rank test to assess whether observed heterozygosity exceeded that expected at mutation-drift equilibrium because the test is robust for small sample size (<30) and a small number of loci (<20) [66].

To examine whether gene flow may be caused by first generation (*F_0_*) immigrants from unsampled populations, we used the Bayesian assignment procedure of Rannala and Mountain [71], as implemented in Geneclass v. 2.0 [72]. This procedure uses the *L*_h_/*L*_max_ likelihood test statistic to identify migrants with an alpha level of 0.01 [71].

## Results

### Population molecular variation

Bachman's Sparrows (n = 226) from 11 different sampling sites were genotyped at 19 microsatellite loci (Table S1). One locus (Zole F11) was dropped because results suggested the presence of null alleles and consistent deviations from HWE across populations. After Bonferroni correction, significant deviations from HWE (p<0.05) were found for three loci: Am 08, Am 18 and Am 20; however, the deviations were not consistent across populations, so these loci were kept for subsequent analysis. Linkage disequilibrium was observed for Aca 01 and Aca 17, and Asμ09 and Zole E11, but the associations were not present in all populations, suggesting the loci were not linked. Individual loci were polymorphic with 2–60 alleles per locus. Average allelic richness was 8.16 (Table 2). Average expected heterozygosity was similar among populations, and in all but North Carolina, the average observed heterozygosity was slightly lower than average expected heterozygosity (Table 2). The inbreeding coefficient *F_IS_* ranged from −0.0130 to 0.0678 and was positive in all but the North Carolina population (Table 2).

**Table 2:** Genetic variation in 11 Bachman's Sparrow populations.

Population	*n*	*H_O_*	*H_E_*	*A*	*AR*	*AR*	*F_IS_*
					(populations with n<10)	(populations with n>10)	
Abita Springs	15	0.7320 (±0.2703)	0.7664 (±0.2435)	9.167	3.006	8.246	0.0447
Avon Park Air Force Range	47	0.7524 (±0.2292)	0.7801 (±0.2342)	14.556	3.047	8.540	0.0355
Dry Prong	25	0.7358 (±0.2537)	0.7716 (±0.2549)	11.722	3.042	8.543	0.0470
Fort Polk	25	0.7307 (±0.2838)	0.7740 (±0.2481)	11.556	3.043	8.426	0.0572
Kisatchie National Forest	15	0.7199 (±0.2347)	0.7563 (±0.2518)	8.722	2.971	7.818	0.0489
North Carolina	3	0.7222 (±0.3284)	0.7148 (±0.3015)	3.667	2.822	-	−0.0130
Palustris Experimental Forest	13	0.7279 (±0.2744)	0.7789 (±0.2356)	8.778	3.046	8.236	0.0678
Sandy Hollow	29	0.7148 (±0.2762)	0.7522 (±0.2706)	10.778	2.980	8.066	0.0494
Tall Timbers Research Station	33	0.7314 (±0.2484)	0.7732 (±0.2502)	12.333	3.039	8.343	0.0529
Talisheek Pine Wetland Preserve	7	0.7460 (±0.2477)	0.7807 (±0.2361)	6.444	3.044	-	0.0489
Camp Whispering Pines	14	0.7145 (±0.2600)	0.7425 (±0.2211)	7.889	2.885	7.234	0.0394
**Mean**		**0.7298**	**0.7628**	**9.601**	**2.993**	**8.161**	**0.0435**

Abbreviations given for sample size (n), observed heterozygosity (H_O_; mean ± std. error), unbiased expected heterozygosity (H_E_; mean ± std. error), average number of alleles/locus (A), allelic richness (AR), and inbreeding coefficient (F_IS_).

**Figure 3:**
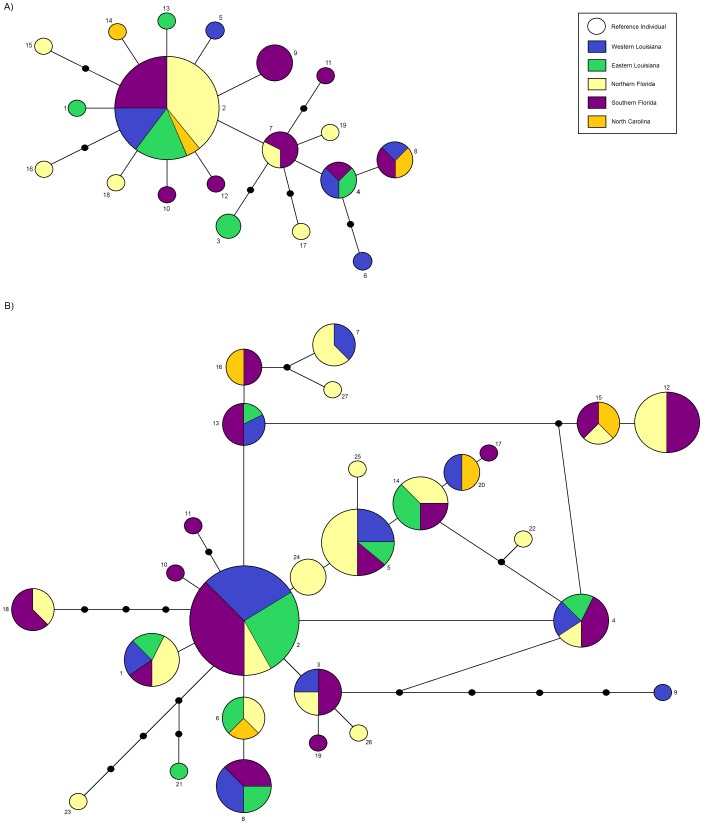
Unrooted parsimony haplotype networks for five regional populations of Bachman's Sparrow. A) mitochondrial ND2 sequence data, and; B) nuclear TGFβ2 sequence data. Areas of circles are proportional to the number of individuals with that haplotype and haplotype number is listed next to circles. A haplotype found in a single individual is given as a size reference in the legend. Small black circles indicate a missing haplotype (one that either was not recovered during sampling or is extinct).

DNA sequence analysis produced 19 haplotypes at ND2 and 27 haplotypes at TGFβ2 after data were phased (Figure 3a & b). Overall sequence diversity within populations was low with nucleotide diversity (π) ranging from 0.0015 to 0.0026 for ND2 and 0.0044 to 0.0076 for TGFβ2 (Table 3). Sequence divergence between regional populations was also low for both genes (Table 4). Despite low nucleotide diversity, both loci had multiple haplotypes within individual populations and high haplotype diversity that ranged from 0.692 to 1.000 for ND2 and 0.925 to 1.00 for TGFβ2 (Table 3).

**Table 3:** Genetic diversity at ND2 and TGFβ2 for five regional Bachman's Sparrow populations including sample size (n), nucleotide diversity (π), number of haplotypes, and haplotype diversity with standard deviation.

	ND2				TGFβ2			
Population Grouping	*n*	π	# of Haplotypes	Haplotype Diversity	*n*	π	# of Haplotypes	Haplotype Diversity
Western Louisiana	7	0.0022	5	0.857 (±0.137)	7	0.0050	10	0.925 (±0.047)
Eastern Louisiana	8	0.0021	5	0.857 (±0.108)	6	0.0044	9	0.939 (±0.058)
Northern Florida	14	0.0015	7	0.692 (±0.137)	14	0.0067	17	0.960 (±0.019)
Southern Florida	15	0.0017	8	0.867 (±0.067)	13	0.0055	16	0.945 (±0.027)
North Carolina	3	0.0026	3	1.000 (±0.272)	2	0.0076	4	1.000 (±0.177)

The western Louisiana grouping includes Fort Polk WMA, Dry Prong, Kisatchie National Forest and Palustris Experimental Forest sampling locations. The eastern Louisiana grouping includes Camp Whispering Pines, Sandy Hollow WMA, Abita Springs, Talisheek Pines Wetlands Preserve and Lee Memorial Forest. Northern Florida is the Tall Timbers Research Station and southern Florida is the Avon Park Air Force Range.

**Table 4:** Estimates of mitochondrial (ND2) and nuclear (TGFβ2) DNA sequence divergence between five regional Bachman's Sparrow populations.

	ND2					TGFβ2				
	Western Louisiana	Eastern Louisiana	Northern Florida	Southern Florida	North Carolina	Western Louisiana	Eastern Louisiana	Northern Florida	Southern Florida	North Carolina
**Western Louisiana**		0.00003	0.00072	−0.00002	−0.00037		−0.00017	0.00002	−0.00009	−0.00049
**Eastern Louisiana**	0.00217		0.00005	0.00003	−0.00015	0.00452		0.00012	0.00002	−0.00034
**Northern Florida**	0.00196	0.00061		0.00002	−0.00011	0.00590	0.00566		0.00002	−0.00057
**Southern Florida**	0.00192	0.00053	0.00159		0.00084	0.00517	0.00495	0.00613		−0.00057
**North Carolina**	0.00202	0.00217	0.00190	0.00075		0.00584	0.00566	0.00661	0.00600	

The number of net nucleotide substitutions per site between populations (*D_a_*) is located above the diagonal. The average number of nucleotide substitutions per site between populations (*D_xy_*) is located below the diagonal. The western Louisiana grouping includes Fort Polk WMA, Dry Prong, Kisatchie National Forest and Palustris Experimental Forest sampling locations. The eastern Louisiana grouping includes Camp Whispering Pines, Sandy Hollow WMA, Abita Springs, Talisheek Pines Wetlands Preserve and Lee Memorial Forest. Northern Florida is the Tall Timbers Research Station and southern Florida is the Avon Park Air Force Range.

### Analyses of population genetic structure

Global *F_ST_* was 0.012 (±0.002) for microsatellite data, indicating slight genetic structure. Small but significant differences in pairwise *F_ST_* were detected for approximately half of the sampled populations, with values ranging from 0.0001 to 0.0574 (Table 5). *R_ST_* ranged from −0.0003 to 0.1893 (Table 5). Pairwise *F_ST_* and *R_ST_* indicated that genetic differentiation was lowest between Fort Polk Wildlife Management Area and both Kisatchie National Forest and Palustris Experimental Forest, whereas samples collected from North Carolina and Camp Whispering Pines were the most genetically differentiated (Table 5). Camp Whispering Pines was divergent from most populations with the highest significant pairwise *F_ST_* and *R_ST_* estimates for 10 and 8 population pairs, respectively (Table 5).

**Table 5:** Pairwise estimates of F_ST_ (below diagonal) and R_ST_ (above diagonal) for eleven populations, arranged from west to east.

	DP	FP	KNF	PEF	WP	SH	TNC	AS	TTRS	AP	NC
**DP**		−0.0058	−0.0003	−0.0106	0.0915	0.0301	0.0082	−0.0164	0.0042	−0.0039	−0.0410
**FP**	0.0007		0.0058	0.0060	**0.0968**	**0.0311**	0.0171	−0.0052	**0.0105**	−0.0005	−0.0498
**KNF**	0.0063	0.0001		0.0047	0.0390	0.0126	0.0505	−0.0116	−0.0042	0.0108	−0.0165
**PEF**	0.0026	0.0001	0.0029		**0.1348**	0.0318	0.0603	−0.0130	0.0076	−0.0010	−0.0232
**WP**	**0.0342**	**0.0255**	**0.0255**	**0.0231**		**0.0614**	**0.1893**	**0.0920**	**0.0892**	**0.1185**	**0.1584**
**SH**	**0.0098**	**0.0062**	**0.0137**	0.0081	**0.0332**		**0.1264**	−0.0000	**0.0314**	**0.0483**	−0.0032
**TNC**	0.0108	0.0063	0.0101	0.0035	**0.0422**	**0.0160**		0.0401	**0.0604**	0.0212	0.0077
**AS**	0.0038	0.0002	0.0091	0.0027	**0.0391**	**0.0130**	0.0108		−0.0029	−0.0036	−0.0432
**TTRS**	**0.0095**	0.0021	**0.0110**	0.0051	**0.0364**	**0.0138**	0.0092	0.0069		0.0050	−0.0291
**AP**	**0.0113**	0.0032	**0.0139**	**0.0067**	**0.0347**	**0.0188**	**0.0188**	**0.0132**	0.0018		−0.0505
**NC**	0.0153	0.0115	0.0306	0.0162	**0.0574**	0.0208	0.0183	0.0209	0.0110	0.0047	

Significant p-values (p≤0.05) indicated in bold. DP  =  Dry Prong, FP  =  Fort Polk, KNF  =  Kisatchie National Forest, PEF  =  Palustris Experimental Forest, WP  =  Camp Whispering Pines, SH  =  Sandy Hollow, TNC  =  Talisheek Pine Wetlands Preserve, AS  =  Abita Springs, TTRS  =  Tall Timbers Research Station, AP  =  Avon Park, NC  =  North Carolina.

AMOVA results suggested that no population structure existed for either nuclear (p = 0.926±0.021) or mitochondrial sequences (p = 0.250±0.096; Table 6). Nearly all the genetic diversity in sequence data was attributed to within-population variation: 95.07% from mitochondrial (ND2) haplotypes and 103.41% from nuclear (TGFβ2) haplotypes (Table 6). Values >100% can occur when there is no genetic structure and the estimated parameter is zero [73].

**Table 6:** AMOVA results using mitochondrial ND2 and nuclear TGFβ2 sequences from five regional Bachman's Sparrow populations.

Source of Variation	d.f.		Sum of Squares		Variance Components		Percentage of Variation	
	TGFβ2	ND2	TGFβ2	ND2	TGFβ2	ND2	TGFβ2	ND2
Among Groups	4	4	5.518	1.543	0.0870	−0.0552	5.40	−13.45
Among Populations Within Groups	6	7	7.125	3.571	−0.1419	0.07532	−8.82	18.47
Within Populations	75	35	124.833	13.567	1.6644	0.38762	103.41	95.07
Total	85	46	137.477	18.681	1.6095	0.40772		

**Figure 4:**
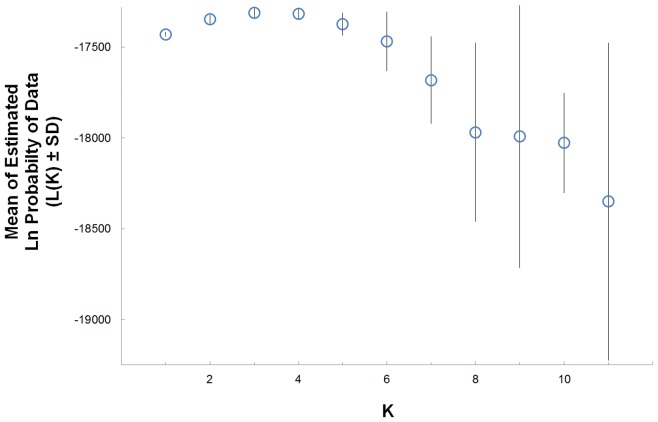
Mean of estimated ln probability of data using LocPrior in Structure for K = 1–11. Using Structure Harvester, the most likely K = 2.

**Figure 5:**
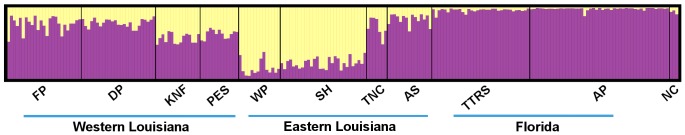
STRUCTURE plot with LocPrior for K = 2 populations. Each column represents an individual, each color denotes a population cluster. Population abbreviations are as follows: Abita Springs (AS), Avon Park (AP), Dry Prong (DP), Fort Polk WMA (FP), Kisatchie National Forest (KNF), North Carolina (NC), Palustris Experimental Forest (PEF), Sandy Hollow WMA (SH), Tall Timbers Research Station (TTRS), Talisheek Pine Wetlands Preserve (TNC), Camp Whispering Pines (WP).

Structure in combination with the method of Evanno et al. [54] suggested two population clusters. K = 2 had the highest mean LnP(K) (−17338.8) and delta K value (11.7) without the LocPrior algorithm. With the LocPrior algorith, K = 3 had the highest mean LnP(K) (−17312.5 versus −17346.8 for K = 2; Figure 4), but K = 2 retained the highest delta K value (1.9 versus 1.4 for K = 3). Of the two population clusters, one included two of the four eastern Louisiana sites and the sites in Florida and North Carolina (Figure 5). The second cluster consisted of the remaining populations in eastern Louisiana (Figure 5). All remaining populations appeared to be a mixture of the two clusters (Figure 5). The average value of *r* for 25 runs of *K* = 2 was 0.73, indicating that location and genotype data were more informative in inferring ancestry than genotype data alone. Structure Harvester results are based on changes in the average likelihood score (*ΔK*) where estimates for *K* = 1 cannot be calculated.

**Figure 6:**
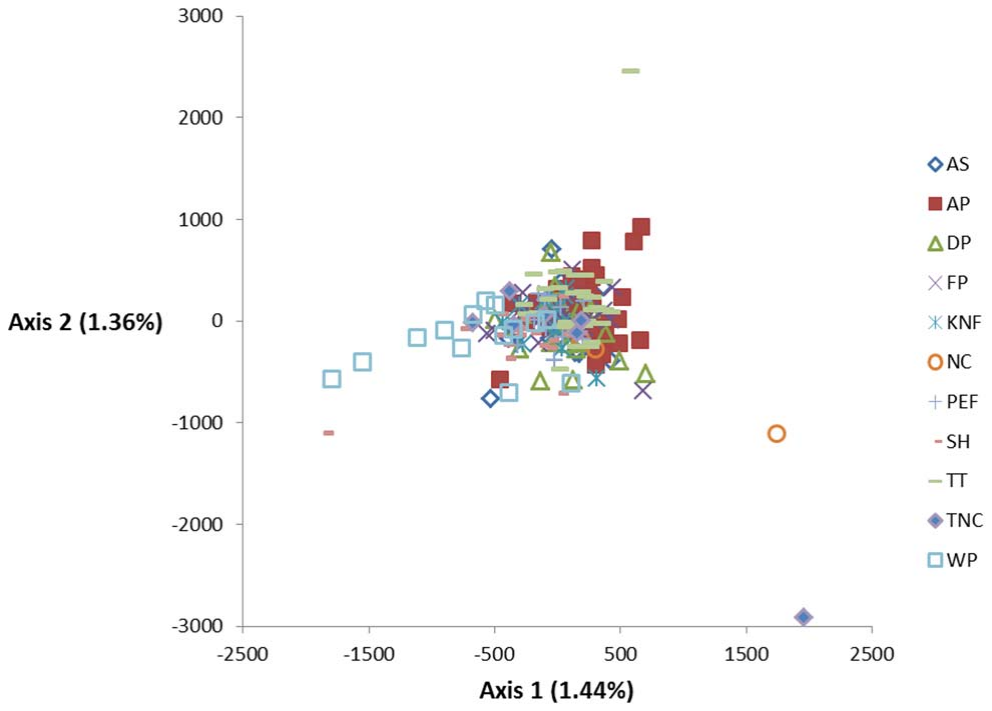
Factorial correspondence analysis of 226 Bachman's Sparrow individuals from eleven study populations. Population abbreviations are as follows: Abita Springs (AS), Avon Park (AP), Dry Prong (DP), Fort Polk WMA (FP), Kisatchie National Forest (KNF), North Carolina (NC), Palustris Experimental Forest (PEF), Sandy Hollow WMA (SH), Tall Timbers Research Station (TTRS), Talisheek Pine Wetlands Preserve (TNC), Camp Whispering Pines (WP).

**Figure 7:**
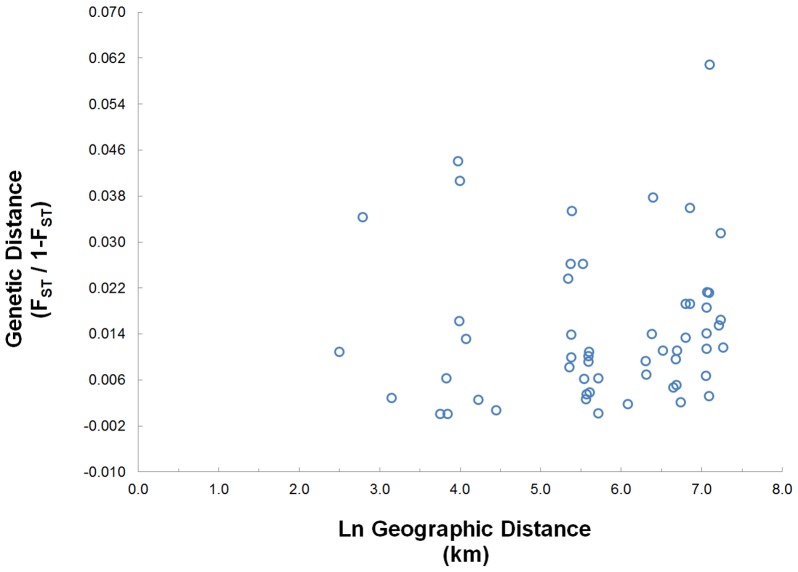
Isolation by distance between pairwise genetic versus pairwise geographical distances. Analyses used a reduced major axis regression (r^2^ = 0.006, intercept  = −0.041±0.008, p = 0.226) calculated from a Mantel test (r = 0.076, *p* = 0.314).

Geneland and FCA results suggested a single population. FCA analysis explained only 2.80% of the variation among individuals and produced no discernible separation among geographic areas (Figure 6). Geneland results suggested a single population with no barriers to gene flow as given by a map of posterior probability (not shown). Finally, the isolation-by-distance analysis showed no significant relationship between geographic distance and genetic distance (Figure 7; r^2^ = 0.006, intercept = −0.041±0.008, p = 0.226) and there was no relationship between geographic distance and genetic distance matrices based on the Mantel test (r = 0.076, p = 0.314).

Sequence data suggested that several populations had unique haplotypes (Figures 3a & b). ND2 sequences consisted of 19 haplotypes (GenBank accession numbers KJ880978–KJ880996), with 15 (83%) of the haplotypes unique to particular regional populations (Figure 3a; KJ880978, KJ880979, KJ880982–KJ880984, KJ880986–KJ880988, KJ880990–KJ880996). The most common haplotype overall (KJ880989) was shared by 42.5% of the 47 individuals sampled. The highest frequency of a single, unique haplotype (KJ880986) occurred in south Florida, and was present in three (6.4%) of the 47 individuals. Similar structure was found with nuclear sequence data (TGFβ2; Figure 3b). There were 27 haplotypes (GenBank accession numbers KM056981–057007), including 11 (40.7%) unique to particular populations (KM056989–KM056991, KM056997, KM056999, KM057001–KM057003, KM057005–KM057007). The most common haplotype was shared by 30.2% of the 43 individuals sampled (KM056982). Despite the presence of private haplotypes, there was no clear geographical pattern in their distribution. The parsimony tree for both ND2 and TGFβ2 was star-like (Figures 3a & b). Neighbor joining trees using ND2 sequence data produced a tree with no clear geographic pattern. The neighbor joining tree built with TGFβ2 sequence data produced a single unresolved polytomy. Polytomies can suggest multiple, simultaneous speciation events, but in this case the tree is probably caused by reduced resolution created by the low number of polymorphic sites. Both neighbor joining trees suggested little, if any, genetic differentiation among the sampled populations.

### Bottlenecks and Population Connectivity

Excess heterozygosity indicative of population bottlenecks was observed in four populations: Fort Polk (p = 0.037), North Carolina (p = 0.025), Talisheek Pine Wetlands Preserve (p = 0.049), and Camp Whispering Pines (p = 0.030), but small samples for Talisheek Pine Wetlands Preserve and North Carolina could produce false positives. False positives can also be observed in populations experiencing high rates of migration (Pope et al. 2000), which may be relevant here. Using Geneclass, we detected 15 first generation (*F_0_*) migrants that were assigned to areas other than their sampling location (Table 7); however, Geneclass does not perform well when population differentiation is slight, so these migrants may simply reflect individuals with rare alleles or individuals from unsampled populations.

**Table 7:** Results of migrant detection analysis in GENECLASS showing individuals with significant assignment probabilities (p<0.01) for population origins other than the study site in which they were sampled.

Sample	Geographic origin	Geneclass locality of highest probability assignment	Geneclass highest assignment probability
LSUMZ 2470	Abita Springs	Kisatchie National Forest	0.0026
11009	Avon Park	Fort Polk	0.0039
11011	Avon Park	Tall Timbers	0.0041
58407	Fort Polk	Kisatchie National Forest	0.0096
58481	Fort Polk	Abita Springs	0.0069
58497	Dry Prong	Sandy Hollow	0.0098
58428	Kisatchie National Forest	Avon Park	0.0012
58429	Kisatchie National Forest	Palustris Experimental Forest	0.0022
58468	Sandy Hollow	Fort Polk	0.0094
07738	Tall Timbers	Sandy Hollow	0.0039
07813	Tall Timbers	Avon Park	0.0046
47760	Tall Timbers	Abita Springs	0.0061
58450	Talisheek Pine Wetlands	Palustris Experimental Forest	0.0077
58447	Camp Whispering Pines	Kisatchie National Forest	0.0034
58448	Camp Whispering Pines	Fort Polk	0.0019

## Discussion

We examined genetic structure and diversity in Bachman's Sparrow to assess the potential effects of large natural barriers, such as the Mississippi River, and recent habitat loss and fragmentation. Most of our analyses showed high genetic diversity (Table 2), little to no inbreeding (Table 2), and weak genetic population structure (Tables 4, 5 & 6, Figures 3 & 6) for both microsatellite and sequence data. Our results suggest a single, panmictic population with considerable gene flow among subpopulations. The virtual absence of genetic structure across such a large area was contrary to predictions based on existing subspecific designations, the patchy distribution of longleaf pine savannahs in which Bachman's Sparrow primarily occur, and the widely presumed low dispersal rates of non-migratory Bachman's Sparrow populations [74].

Our sampling areas overlapped broadly with the distribution of non-migratory populations in the southern half of the species' range [74]. These putatively sedentary populations might be expected to show genetic structure over large spatial scales as do sedentary southern populations of House Wren (*Troglodytes aedon*), which have lower genetic diversity and less population structure than northern populations with seasonal north-south migrations [75]. However, our results are more consistent with migratory passerines that have high levels of gene flow even among distantly located populations [76]. For example, genetic differentiation is both small and non-significant among fragmented populations of Brewer's Sparrow (*Spizella breweri*) [77], Reed Buntings (*Emberiza schoeniculus*) [78], and Cerulean Warblers (*Setophaga cerulea*) [79], species that have either north-south or east-west patterns of seasonal migration.

In Bachman's Sparrow, low differentiation and weak population structure (Tables 4, 5 & 6, Figures 3 & 6), and no evidence of isolation-by-distance (Figure 7) suggest significant connectivity among populations across the sparrow's range, at least historically. For example, pairwise *F_ST_* values were low and non-significant for the most distant populations sampled on Fort Polk, Louisiana and North Carolina (∼1,500 km), located at the western and eastern extremes of the range (Table 5). Non-significance may be attributed to low sample size in the North Carolina population (Table 1), but similarly low pairwise *F_ST_* values were observed between Fort Polk and Avon Park, which have large sample sizes and are separated by similar distances (∼1,200 km; Table 5). Overall, differentiation among many sampling locations was significant, but *F_ST_* values were generally low (Table 5). In Louisiana, low pairwise *F_ST_* values (Table 5) and the absence of population differentiation in multiple analyses (Figures 3 & 6) of populations east and west of the Mississippi River also suggest the absence of genetic structure. The break in habitat created by the Mississippi River and habitat fragmentation does not appear to hamper dispersal.

The only evidence we found for any genetic structure in Bachman's Sparrows appeared in our Structure analyses where two populations (Sandy Hollow WMA and Camp Whispering Pines; Figure 5) located closer to the center of the species' range in southeastern Louisiana, clustered separately from the other populations. In this part of the range, considerable sparrow habitat has been lost or degraded by human land-use changes or fire suppression, which has significantly reduced or completely eliminated contiguous forest cover. The higher level of differentiation observed for these populations may indicate that small, isolated fragments of habitat have detrimental effects on gene flow. However, the inference of two population clusters could also be explained by the reduced precision of Structure and Structure Harvester when F_ST_ values are low [80].

Dispersal, which may account for low levels of genetic differentiation, has not been extensively studied in Bachman's Sparrows, but there are indications that the sparrows are able to travel large distances. First, northern populations are migratory and move south from North Carolina, Kentucky, and Arkansas to southern Florida and westward into the Gulf States [74]. Second, Bachman's Sparrows greatly expanded their range north into Pennsylvania and Illinois during the early 1900s (see below) [24], [81], [82]. Bachman's Sparrows have also been observed using clearcuts and utility right-of-ways [74], suggesting that this species has greater mobility than assumed in some studies [83], [84]. Finally, individuals have been observed establishing new territories or re-establishing and defending previously held territories immediately following fire [20], [30], [85], [86] (personal field observation).

High vagility as an adaptation to ephemeral habitat is consistent with the lack of genetic structure observed in our study. Bachman's Sparrow habitat suitability is closely linked to ground-cover conditions, and individuals typically abandon areas that have not been burned every 2–3 years [24]–[28]. Historically, longleaf pine forests burned frequently with fire-return intervals averaging <3 years [25], and fires certainly occurred at much larger scales than current prescribed fires. Fires likely produced large gaps among unburned fragments [25] leading to a matrix of suitable, recently burned habitat and unsuitable, overgrown habitat, a habitat matrix that has probably existed on the landscape for a long time. The estimated generation length for Bachman's Sparrows [87] is usually greater than the average fire-return intervals recorded historically, so high dispersal rates may be an adaptation that enables individuals to colonize ephemeral habitat [26]. Indeed, similar instances of apparent genetic connectivity and weak genetic structure have been observed in other avian species associated with longleaf pine forests (e.g. Red-cockaded Woodpeckers, *Picoides borealis*) [88]–[90] as well as avian species in Australia that are adapted to landscapes frequently fragmented by fire (e.g. Mallee Emu-wren, *Stipiturus mallee*) [20]. These empirical results are also supported by several modeling studies, which have suggested that higher dispersal capability should be maintained in species occupying landscapes that have frequent temporal and spatial changes whereas species found in less disturbed and more contiguous habitat should have less pronounced dispersal capability [91], [92].

Although high vagility as an adaptation to ephemeral habitat may explain weak population structure on a local scale, it is still surprising to see weak population structure among distant populations with different subspecific designations: Bachman's Sparrows probably do not need to travel thousands of km to find suitable habitat. Accordingly, weak population structure between distant populations may be the product of range expansion and retraction. During the early 1900s, Bachman's Sparrow moved northward and occupied suitable habitat on abandoned farms and fallow pastures from Pennsylvania to Illinois, which mimicked the savannah-like understory of southern pine forests [24], [81], [82]. The range retracted as agricultural practices changed and farmlands became more urbanized [24], [81], [82], [93]. During range expansion, individuals from distinct populations may have bred together, homogenizing genetic variation. If offspring of mixed genetic descent returned south or genetically distinct individuals returned to a population other than their population of origin, any population structure that existed in the past may have been eliminated. Current populations might exhibit low differentiation because genetic drift, selection, and mutation have not had sufficient time to produce differences among populations [20]. An examination of Bachman's Sparrow historic genetic variation prior to the range expansion and more extensive sampling across Bachman's Sparrow populations should provide insight on this possibility.

### Implications for Conservation

Low genetic differentiation among Bachman's Sparrow populations suggests that neither natural barriers nor anthropogenic fragmentation has caused population differentiation, loss of genetic diversity, or inbreeding. The current lack of differentiation across the species' geographic range means that recognition of distinctive subspecies may not be necessary for management purposes. However, an examination of historical genetic variation may be necessary to confirm this conclusion because any genetic structure that was formerly present may have been weakened by range expansion and contraction. Furthermore, distinct populations identified by plumage differences as described in Sibley (2000) and the AOU [21] may be linked to genes that we did not assess. More comprehensive genome-scale studies will be needed to assess this possibility. Given high levels of diversity, low levels of inbreeding, and apparent panmixia, translocations to provide gene flow among populations and counteract the negative effects of genetic drift and inbreeding depression do not appear to be necessary. Although our results imply that habitat fragmentation and loss had little effect on the erosion of genetic diversity of Bachman's Sparrow populations, it is still important to consider the effects that isolation may have on the management of this species. High vagility may be an adaptation to ephemeral habitat, but Bachman's Sparrow populations nevertheless require sizeable blocks of suitable habitat to persist over the long term.

## Supporting Information

Table S1 **Characteristics of 23 microsatellite loci screened in Bachman's Sparrows.**
(DOCX)
