# Supplementary material for: Adaptation to Ephemeral Habitat May Overcome Natural Barriers and Severe Habitat Fragmentation in a Fire-Dependent Species, the Bachman's Sparrow (Peucaea aestivalis)
Source: PLoS One. 2014 Sep 2;9(9):e105782. doi: 10.1371/journal.pone.0105782 (PMC4152175; doi:10.1371/journal.pone.0105782)
Supplement: Table S1 — Characteristics of 23 microsatellite loci screened in Bachman's Sparrows. (DOCX) [file pone.0105782.s001.docx]

| **Table S1.** Characteristics of 23 microsatellite loci screened in Bachman’s Sparrows. Annealing temperature in degrees Celsius (*T_A_*), size range of alleles (bp), and number of alleles (*N_A_*). * denotes loci used in final analyses. | | | | | | | |
| --- | --- | --- | --- | --- | --- | --- | --- |
| Locus | Repeat Motif | Primer sequence | *T_A_* | Size range (bp) | *N_A_* | GenBank  accession no. | Reference |
| *Aca 01 | (TCTA)_14_(TCA)_2_-TCTATCA(TCTA)_13_ | F: AGCCCACTAATGGGTTTTCC | 58 | 164-224 | 24 | EF447093 | [[1](#_ENREF_1)] |
|  |  | R: TGAGTGTTCAAAGTTGCCAGA |  |  |  |  |  |
| *Aca 05 | (TGTC)_2_(TATG)_7_-(TATC)_14_ | F: CCTGCTAGGCTGCATCTTCT | 58 | 204-306 | 40 | EF447095 | [[1](#_ENREF_1)] |
|  |  | R: GAGTGTCATCACATTTGTACTTTGG |  |  |  |  |  |
| *Aca 17 | (TCTA)_13_(TC)_9_ | F: GGAGCATGTGACAATGGAGT | 58 | 251-339 | 23 | EF447100 | [[1](#_ENREF_1)] |
|  |  | R: TCTGTGCTGTTCCAAGCAGA |  |  |  |  |  |
| *Am 02 | (CTCA)_13_ | F: CTGCAAAATGTTCAGGCC | 58 | 246-262 | 5 | JQ845066 | [[2](#_ENREF_2)] |
|  |  | R: GTTTACTGGAACCTTGCATGCAAC |  |  |  |  |  |
| *Am 08 | (AGGT)_13_ | F: GTTTGGGACATGAAAAGCTGGCAG | 58 | 212-354 | 60 | JQ845069 | [[2](#_ENREF_2)] |
|  |  | R: GGTCATCGGTGGGTTG |  |  |  |  |  |
| *Am 12 | (AGAT)_15_ | F: GTTTCCCCACCCATTTTCACCATC | 58 | 239-411 | 34 | JQ845070 | [[2](#_ENREF_2)] |
|  |  | R: GAACTTCCAAACACAAAGGC |  |  |  |  |  |
| *Am 14 | (ATAG)_10_ | F: GACCTGCAAGAGAGGTGTC | 58 | 141-145 | 2 | JQ845071 | [[2](#_ENREF_2)] |
|  |  | R: GTTTAGTTGAGTTGTTTGATCCAGGC |  |  |  |  |  |
| *Am 18 | (ATAG)_15_ | F: GTTTCACCAGGAAACCCTTGCAAC | 58 | 146-260 | 18 | JQ845073 | [[2](#_ENREF_2)] |
|  |  | R: GTCTCTGCCTGCATCTTCAG |  |  |  |  |  |
| *Am 20 | (ATAG)_12_...-(AGAC)_5_ | F: GTTTGGCTTTTCAAGGGTCTGTCC | 58 | 156-296 | 35 | JQ845074 | [[2](#_ENREF_2)] |
|  |  | R: AACCCCAACCTGTCCCATG |  |  |  |  |  |
| Ase64 | (AGGG)_9_(ATGG)_12_ | F: CCACCTTTCATACTGGGGAG | 48 | 276–300 | 5 | AJ276793 | [[3](#_ENREF_3)] |
|  |  | R: TTCAGCCAGTCAGTGTAGCC |  |  |  |  |  |
| *Asµ09 | (CA)_25_ | F: CTTTGATTACAGAAATATGTCTTCT | 48 | 137-161 | 11 | AY172992 | [[4](#_ENREF_4)] |
|  |  | R: GAAAGAGGCATGCTCGTAT |  |  |  |  |  |
| Asµ18 | (AC)_7_TC(AC)_9_ | F: ACACAGAGAGACACAAATTCAT | 48 | 144-152 | 9 | AY172994 | [[4](#_ENREF_4)] |
|  |  | R: AAATGCTACTGAGGTAAAGTCC |  |  |  |  |  |
| *FhU2 | (CT)_12_ | F: GTGTTCTTAAAACATGCCTGGAGG | 48 | 144-184 | 18 | X84361 | [[5](#_ENREF_5)] |
|  |  | R: GCACAGGTAAATATTTGCTGGGCC |  |  |  |  |  |
| LEI 160 | (AG)_13_ | F: CAAAGTAATCAGCTTGTGCTAC | 58 | 248-254 | 2 | X85523 | [[6](#_ENREF_6)] |
|  |  | R: CATTTCCACCGCATTGAGCAG |  |  |  |  |  |
| *Mme 12 | (CCCACA)_13_ | F: AGGGACTGTCACTGTGGGACTGAAG | 48 | 199-203 | 2 | AF127385 | [[7](#_ENREF_7)] |
|  |  | R: TGGCTTTATGGAACAAGGCATC |  |  |  |  |  |
| *SOSP 01 | (GGAT)_17_GCAT- (GGAT)_2_ | F: GCCAACACCCTCAACAAGAT | 48 | 219-259 | 11 | GU301255 | [[8](#_ENREF_8)] |
|  |  | R: ACCAACTGATGCACCTTCTG |  |  |  |  |  |
| *SOSP 02 | (CTGT)6(GT)3 | F: AAACTCGCGTCTTTGCTAGG | 48 | 179-219 | 18 | GU301256 | [[8](#_ENREF_8)] |
|  |  | R: CAGGTGTCCTGCAGATGTTG |  |  |  |  |  |
| *SOSP 04 | (TGTC)_6_ | F: GGTTGATGGGGATGTTTCTG | 48 | 186-232 | 22 | GU301258 | [[8](#_ENREF_8)] |
|  |  | R: CTTCTTGAGCTTGGGGTCAC |  |  |  |  |  |
| *SOSP 14 | (CTAT)_16_ | F: GGGCTTTCTGGCAAAGATATG | 48 | 187-287 | 33 | GU301268 | [[8](#_ENREF_8)] |
|  |  | R: AAAAAGGGGCTTAGGTCCAG |  |  |  |  |  |
| *Zole C11 | (ATCT)_14_ | F: TCCATGCTTCTGAACTGCC | 58 | 149-203 | 16 | EU410392 | [[9](#_ENREF_9)] |
|  |  | R: ACACCTGCTTTTCCTGACTG |  |  |  |  |  |
| *Zole E11 | (ATCT)_13_ | F: AGAATGCTCTGGAACCGGC | 58 | 175-219 | 18 | EU410395 | [[9](#_ENREF_9)] |
|  |  | R: AGGACCTGTGTGCCAATTAAG |  |  |  |  |  |
| Zole F11 | (ATCC)_10_ | F: AACCAAGCCACCACAATGC | 58 | 232-336 | 25 | EU410397 | [[9](#_ENREF_9)] |
|  |  | R: GACAGGCACTAGGATGGGAG |  |  |  |  |  |
| Zole G03 | (AGAT)_12_ | F: GCCACGTTACACATCCTGC20 | 60 | 214-271 | 16 | EU410398 | [[9](#_ENREF_9)] |
|  |  | R: CTGGCATTCCAAAGCTGGG |  |  |  |  |  |

**Literature Cited**

1. Hill CE, Tomko S, Hagen C, Schable NA, Glenn TC (2008) Novel microsatellite markers for the saltmarsh Sharp-tailed Sparrow, *Ammodramus caudacutus* (Aves: Passeriformes). Molecular Ecology Resources 8: 113-115.

2. Lehmicke AJJ, Berry BE, Shamblin BM, Lennon DM, Woodrey MS, et al. (2012) Isolation and characterization of tetranucleotide microsatellite loci from the Seaside Sparrow (*Ammodramus maritimus*). Conservation Genetics Resources 4: 881-884.

3. Richardson DS, Jury FL, Dawson DA, Salgueiro P, Komdeur J, et al. (2000) Fifty Seychelles Warbler (*Acrocephalus sechellensis*) microsatellite loci polymorphic in Sylviidae species and their cross-amplification in other passerine birds. Molecular Ecology 9: 2226-2231.

4. Bulgin NL, Gibbs HL, Vickery P, Baker AJ (2003) Ancestral polymorphisms in genetic markers obscure detection of evolutionarily distinct populations in the endangered Florida Grasshopper Sparrow (*Ammodramus savannarum floridanus*). Molecular Ecology 12: 831-844.

5. Primmer CR, Moller AP, Ellegren H (1996) A wide-range survey of cross-species microsatellite amplification in birds. Molecular Ecology 5: 365-378.

6. Crooijmans RPMA, Dijkhof RJM, van der Poel JJ, Groenen MAM (1997) New microsatellite markers in chicken optimized for automated fluorescent genotyping. Animal Genetics 28: 427-437.

7. Jeffery KJ, Keller LF, Arcese P, Bruford WW (2001) The development of microsatellite loci in the Song Sparrow, *Melospiza melodia* (Aves) and genotyping errors associated with good quality DNA. Molecular Ecology Notes 1: 11-13.

8. Sardell RJ, Keller LF, Arcese P, Bucher T, Reid JM (2010) Comprehensive paternity assignment: genotype, spatial location and social status in Song Sparrows, *Melospiza melodia*. Molecular Ecology 19: 4352-4364.

9. Poesel A, Gibbs HL, Nelson DA (2009) Twenty-one novel microsatellite DNA loci isolated from the Puget Sound White-crowned Sparrow, *Zonotrichia leucophrys pugetensis*. Molecular Ecology Resources 9: 795-798.
